# Complement-Dependent Activity of CD20-Specific IgG Correlates With Bivalent Antigen Binding and C1q Binding Strength

**DOI:** 10.3389/fimmu.2020.609941

**Published:** 2021-01-11

**Authors:** Sina Bondza, Anita Marosan, Sibel Kara, Josephine Lösing, Matthias Peipp, Falk Nimmerjahn, Jos Buijs, Anja Lux

**Affiliations:** ^1^ Department of Immunology, Genetics and Pathology, Uppsala University, Uppsala, Sweden; ^2^ Ridgeview Instruments AB, Uppsala, Sweden; ^3^ Department of Genetics, Friedrich-Alexander University, Erlangen, Germany; ^4^ Division of Stem Cell Transplantation and Immunotherapy, Department of Medicine II, UKSH, CAU Kiel, Kiel, Germany

**Keywords:** CD20, C1q, Rituximab, Ofatumumab, Obinutuzumab, CDC, B cells, interaction

## Abstract

Monoclonal antibodies directed against the CD20 surface antigen on B cells are widely used in the therapy of B cell malignancies. Upon administration, the antibodies bind to CD20 expressing B cells and induce their depletion *via* cell- and complement-dependent cytotoxicity or by induction of direct cell killing. The three antibodies currently most often used in the clinic are Rituximab (RTX), Ofatumumab (OFA) and Obinutuzumab (OBI). Even though these antibodies are all of the human IgG1 subclass, they have previously been described to vary considerably in the effector functions involved in therapeutic B cell depletion, especially in regards to complement activation. Whereas OFA is known to strongly induce complement-dependent cytotoxicity, OBI is described to be far less efficient. In contrast, the role of complement in RTX-induced B cell depletion is still under debate. Some of this dissent might come from the use of different *in vitro* systems for characterization of antibody effector functions. We therefore set out to systematically compare antibody as well as C1q binding and complement-activation by RTX, OFA and OBI on human B cell lines that differ in expression levels of CD20 and complement-regulatory proteins as well as human primary B cells. Applying real-time interaction analysis, we show that the overall strength of C1q binding to live target cells coated with antibodies positively correlated with the degree of bivalent binding for the antibodies to CD20. Kinetic analysis revealed that C1q exhibits two binding modes with distinct affinities and binding stabilities, with exact numbers varying both between antibodies and cell lines. Furthermore, complement-dependent cell killing by RTX and OBI was highly cell-line dependent, whereas the superior complement-dependent cytotoxicity by OFA was independent of the target B cells. All three antibodies were able to initiate deposition of C3b on the B cell surface, although to varying extent. This suggests that complement activation occurs but might not necessarily lead to induction of complement-dependent cytotoxicity. This activation could, however, initiate complement-dependent phagocytosis as an alternative mechanism of therapeutic B cell depletion.

## Introduction

Monoclonal antibodies (mAb) applied in the treatment of malignant diseases employ different immune-mediated mechanisms that contribute to their efficacy such as antibody-dependent cellular cytotoxicity (ADCC) (1), antibody-dependent cellular phagocytosis (ADCP) (2), as well as activation of the complement system (3). While ADCC and ADCP are accepted as important mechanisms for successful monoclonal antibody (mAb) therapy, the importance of the complement system for mAb therapy is less clear. The Fc-terminus of antibodies harbors a binding site for the serum protein C1q (4) that activates the classical complement pathway which, through a series of proteolytic cleavage events, leads to deposition of the complement component 3b (C3b) on the surface of opsonized cells. If sufficient amounts of membrane bound C3b accumulate on the target cell, eventually pores, called membrane attack complexes (MAC), are formed by complement proteins C6 through C9 that mediate cell lysis; a process termed as complement-dependent cytotoxicity (CDC) (3). Moreover, membrane-bound complement cleavage products such as C3b or C4b also function as opsonins by interacting with complement receptors on effector cells which results in complement-dependent phagocytosis (CDCP) (5). Effective killing of tumor cells by CDC *in vitro* has been demonstrated, especially for certain anti-CD20 antibodies (6–8), but the contribution of complement to tumor killing *in vivo* is debated (9–15). To date, expression of negative regulators of the complement system (6, 16, 17) and exhaustion of complement components have been described to limit CDC efficacy in the clinic (11, 18).

Mechanistically, binding of C1q occurs preferably to a hexameric formation of IgG Fc-tails (19) and it has been shown that antibodies harboring mutations in the Fc-region that facilitate this arrangement can induce CDC more efficiently (20, 21). Depending on their capacity to cluster CD20 on the cell surface, anti-CD20 antibodies are grouped into type I and type II, the latter not having this ability (22, 23). Type I antibodies like Rituximab (RTX) or Ofatumumab (OFA) have been shown to efficiently activate complement, presumably because clustering facilitates the formation of hexameric IgG-Fc platforms suitable for C1q binding (19, 24). Platform formation could also be supported by type I mAbs acting as molecular seeds that locally increase antibody concentration. In contrast, recruitment of the type II mAb Obinutuzumab (OBI) prevented further binding of mAbs as well as complement components (25) providing an explanation for its reduced capacity to activate complement. With respect to their B cell depleting activity, both type I and type II antibodies are able to induce ADCC as well as ADCP (23). Type II antibodies are, however, more efficient at inducing direct cell death (26, 27). Type I and type II CD20-specific mAbs have also been shown to differ in their capacity to be internalized following interaction with FcγRIIb expressed on B cells (28, 29). The underlying molecular properties for the functional classification into type I and II are still debated but include the binding epitope (30), the elbow hinge angle (31, 32), as well as binding orientation (33) and binding stability of the antibody (34).

Recognition by C1q is the crucial step in activation of the classical pathway of complement and stronger binding of C1q to antibody opsonized cells has been correlated with more efficient target cell lysis (19, 35). However, the parameters involved in the formation of the optimal antibody platform for C1q binding are not completely understood yet. Contradicting observations have especially been made with respect to functionally monovalent antibodies inducing CDC more efficiently than their counterparts with bivalent binding capability (19, 36, 37). For C1q binding to antibody opsonized cells, affinity values in the low nM range have been reported without resolution of the kinetic binding parameters (38). Furthermore, the apparent binding affinity for C1q to monomeric IgG in solution is around 10 µM (39), whereas the binding to larger, clustered immune complexes is known to be in the nM range (40).

In a previous study, we analyzed the binding pattern of RTX, OBI and OFA to Daudi cells by real-time interaction analysis and showed that OFA displayed the highest degree of bivalent binding, followed by RTX and then OBI (41). Consequently, the OFA interaction was less dynamic, *i.e.* OFA showed a slower antibody exchange, possibly caused by a higher fraction of antibodies stabilized by bivalent binding. The degree of bivalent target engagement thus positively correlates with how efficiently these mAbs have been shown to induce CDC *in vitro*. As the notion that bivalent target engagement is beneficial for CDC stands in contrast to observations made with antibodies targeting EGFR (19) and HER2 (37), we set out to develop a real-time binding assay to investigate C1q capture on live cells opsonized with CD20 mAbs. We systematically compared CD20 mAb binding, C1q binding and complement-activation, as well as C3b deposition that can trigger target cell killing independently of MAC formation. Several human lymphoma B cell lines that differ in CD20 expression levels and complement-regulatory proteins as well as human primary B cells were included in our analysis to understand the influence of the model system in the context of complement activation by CD20 mAbs *in vitro*. Our data suggests that CD20 specific mAbs differ in their mode of binding which, in combination with the type of target cell, determines efficacy of CDC *in vitro*.

## Materials and Methods

### Cell Culture and B Cell Isolation

Ramos (ATCC), Daudi (ATCC), P493.6, LCL1.11 (kindly provided by Georg Bornkamm, Helmholtz Zentrum, Munich, Germany) and K562 (kind gift from Dr. Stenerlöw, Uppsala University) were cultured in a humidified incubator at 37°C with 5% CO_2_. For interaction analyses, Ramos and K562 cells were maintained in RPMI 1640 cell medium (Biochrom AG) supplemented with 10% heat-inactivated FBS (Sigma Life Science), 2 mM L-glutamine (Biochrom AG) and 100 µg/ml penicillin-streptomycin (Biochrom AG). Daudi and LCL1.11 cells were cultured in the same medium, but with additional sodium pyruvate (Sigma-Aldrich) added to a final concentration of 1 mM. P493.6 cells were cultured in RPMI 1640 without phenol red (Biowest) with the same supplements as for Daudi cells and in addition 0.1 mM MEM non-essential amino acids (Invitrogen). B cell lines for functional assays were cultured in RPMI 1640 (Gibco) supplemented with 10% heat-inactivated FBS (Pan Biotech), 1 mM sodium pyruvate (Gibco), 100 µg/ml penicillin-streptomycin (C-C-Pro), 2 mM L-glutamine (C-C-Pro) and 0.1 mM MEM non-essential amino acids (Gibco). For isolation of human primary B cells, peripheral blood mononuclear cells were purified from blood cones by density gradient centrifugation. PBMCs were then subjected to MojoSort™ Human B Cell Isolation Kit (BioLegend) according to the manufacturer´s instructions. Healthy human and CLL patient PBMCs were isolated from peripheral blood by density gradient centrifugation and either used immediately or stored at −80°C in FBS containing 10% DMSO until usage.

### Seeding for LigandTracer analysis

Cells were immobilized either on petri dishes (Nunc 263991, ThermoFisher Scientific) or LigandTracer MultiDishes 2x2 (Ridgeview Instruments) for real-time binding assays, essentially as previously published (42). In brief, a biomolecular anchor molecule (BAM) (SUNBRIGHT^®^ OE-040CS, NOF Corporation) was dissolved to 2 mg/ml in ddH_2_O water and circular drops of 400 µl were carefully placed onto the dishes and incubated for 1 h at room temperature. After carefully aspirating the BAM solution, cells suspended in PBS (due to differences in size 7.5*10^6^ cells/ml for Ramos, Daudi, P493.6 and LCL1.11, 2.5*10^6^ cells/ml for K562) were placed onto the BAM coated spots. Human primary B cells were resuspended in RPMI 1640 (supplemented with 1 mM sodium pyruvate, 100 µg/ml penicillin-streptomycin, 2 mM L-glutamine and 100 µM MEM non-essential amino acids) and 2*10^6^ cells and seeded on BAM coated spots. Cells were then incubated for 40 min at room temperature. Cells that did not attach were carefully removed and cell culture medium was added. Seeded cells were kept a humidified incubator at 37°C with 5% CO_2_ and used for experiments the next day.

### Antibodies and Protein Labeling

For real-time experiments Rituximab and Ofatumumab were purchased from Apoteket AB (Sweden) in clinical formulation, Rituximab and Ofatumumab for functional experiments, as well as Obinutuzumab were purchased from the pharmacy of the university hospital of Schleswig-Holstein (Kiel, Germany). Fab fragments were generated using the Pierce Fab Preparation Kit (Thermo Fisher Scientific) following the manufacturer’s instructions, which essentially comprised enzymatic digestion with papain followed by removal of the Fc-part *via* a protein A column. Fab fragmentation was verified by running a non-reducing SDS-PAGE with subsequent Coomassie staining (**Supplementary Figure 1**). After Fab fragmentation, a buffer exchange to either PBS or borate buffer pH 9.2 (the latter if the Fab was to be labeled fluorescently) was performed using a Nap-5 Sephadex G-25 column (Illustra, GE Healthcare). Antibodies were diluted to 2 mg/ml in PBS and mixed with borate buffer pH 9.2 in 1:2 volume ratio for fluorescent labeling. Fluorescein isothiocyanate (FITC) was dissolved in DMSO and 100 ng FITC was added for every µg protein. After incubation at 37°C for 90 min, unconjugated FITC was removed by purification through a Nap-5 column (Illustra, GE Healthcare). Labeled antibodies and Fab fragments were kept at 4°C for short term and at −20°C for long storage. For labeling, human complement component C1q (Merck Millipore) was mixed 5:1 with 0.2 M sodium bicarbonate buffer pH 9. Atto488 was dissolved in DMSO and 0.5 µg for every µg protein was added and incubated for 1 h at room temperature. Excess fluorophore was removed through a Nap-5 column and C1q was stored at 4°C overnight and always used for experiments the following day. Protein concentrations after labeling were measured with NanoPhotometer (Implen P360). For C1q a molar extinction coefficient of 2.742*10^5^ cm^−1^M^−1^ was used.

### Real-Time Cell-Binding Assay

LigandTracer Green (Ridgeview Instruments) was used to study molecular interactions on live cells. The instrument consists of an inclined cell dish holder that rotates during the measurement and a fluorescent detector mounted that records signals from the upper position of the cell dish, thereby avoiding fluorescence from the bulk liquid containing unbound ligand. For binding experiments with labeled antibodies or Fab fragments, at least two positions were measured during each rotation: CD20 expressing cells (Ramos, Daudi, P493.6, LCL1.11 or primary B cells) in the target position and K562 cells that lack CD20 or media only as control for subtraction of background fluorescence. Each full rotation takes 70 s and results in at least one background subtracted data point. Experiments were performed with the cell culture medium used for culturing the CD20 expressing cells. After recording a baseline, FITC-labeled protein was added to initiate the association phase. After recording an association phase, the incubation media was changed to either plain media not containing any ligand or media containing unlabeled ligand to monitor dissociation.

For binding experiments with labeled C1q and unlabeled antibodies, four positions were measured during each rotation with each half of the MultiDish 2x2 containing one spot of CD20 expressing cells and one spot with K562 cells for background correction, resulting in two background corrected binding curves with a data collection frequency of 0.86 min^-1^. Cells were pre-incubated with unlabeled antibody in CO_2_ independent RPMI medium at room temperature at a concentration and time that allowed for binding to reach equilibrium. This incubation solution was used as running buffer for LigandTracer experiments with C1q and, after recording a baseline, labeled C1q was added in three increasing concentrations (1.4 nM, 3.9 nM and 9.6 nM) for the association phase. For the dissociation phase, the incubation solution was exchanged to the same media containing unlabeled antibody to keep the antibody concentration constant during the entire experiment.

### Real-Time Interaction Analysis

Real-time binding data for antibodies and Fab fragments was analyzed with TraceDrawer 1.9 (Ridgeview Instruments) according to the 1:1 binding model. The 1:1 Langmuir model assumes that a reversible binding process between a ligand (L) and a target (T) receptor is characterized by a single association rate constant k_a_ and dissociation rate constant k_d_ (Eq. 1) also referred to as on- and off-rates.

[1] $$\left[L]+[T]\overset{k_{a}}{\underset{k_{d}}{⇄}}[LT\right]$$

The affinity K_D_ is calculated from the ratio of the rate constants (Eq. 2).

[2] $$K_{D}=\frac{k_{d}}{k_{a}}$$

The interaction half-life t_1/2_, *i.e.* the time until half of the bound ligands have dissociated, can be calculated from the off-rate (Eq. 2).

[3] $$t_{1/2}=\frac{\ln(2)}{k_{d}}$$

In real-time interaction analysis, the kinetic parameters are extracted from the non-linearity of the binding signal, B, over time which needs to be proportional to the number of ligand-target complexes formed (Eq. 4).

[4] δBδt=ka·[L]·(Bmax−B)−kd·B

For this type of analysis, the number of targets needs to stay constant during the experiment and, in contrast to end-point measurements, target saturation is not required. Besides the interaction rate constants, also the theoretical signal at target saturation B_max_ is estimated from the binding curve.

Real-time binding data for C1q to antibody opsonized cells was also evaluated with InteractionMap (IM). This analysis searches for 1:1-like interactions in a defined k_a_ and k_d_ parameter space that correspond to the measured binding data when summed up (Eq. 5).

[5] $$MeasuredCurve=\Sigma_{i=1}^{n}\Sigma_{j=1}^{m}[W_{\mathrm{ij}}*CurveComponent(conc,k_{a}^{i},k_{d}^{j})]$$

Each interaction is depicted in an on-off plot and colors are assigned according the weighing factors W_ij_: the more an individual interaction contributes to the measured curve, the warmer the color.

### Human Samples

Human serum samples and peripheral blood for CDC assays were collected from healthy individuals with approved consent. Blood cones used to isolate B cells for interaction analysis were provided by the Department of Transfusion Medicine and Haemostaseology of the University Clinics Erlangen, Germany, with informed consent of the donor and the local ethical committee. PBMC from CLL patients were provided by the Department of Medicine II, Kiel, Germany with informed consent of the donors.

### Complement Dependent Cytotoxicity (CDC) Assay

For analysis of CDC induction and complement C3b deposition 5*10^4^ Ramos, Daudi, P493.6 or LCL1.11 cells or 7.5*10^5^ human PBMCs were resuspended in RPMI 1640 supplemented with 10% FBS, 100 µg/ml penicillin-streptomycin, 2 mM L-glutamine, 1 mM sodium pyruvate and 0.1 mM MEM non-essential amino acids. CD20-specific antibodies (OFA, RTX, or OBI) were added at either 20 µg/ml or 2 µg/ml. Human serum was obtained, stored at −20°C and added to 20% of the reaction volume. Controls included serum heat inactivated at 56°C for 30 min or cells incubated with serum/heat-inactivated serum but no CD20-specific antibodies. Cells were then incubated for 30 min at 37°C. The reaction was stopped by addition of ice-cold PBS supplemented with 10% FBS and 0.05% sodium azide. Cells were then stained with DAPI and anti-C3b-FITC (clone 2C6, Cedarlane) for subsequent flow cytometry analysis. Human primary cells were in addition stained with anti-CD19-PE/Cyanine7 (clone HIB19, Biolegend) to label B cells. Samples were acquired on a FACSCantoII (BD Biosciences) and analyzed using FACSDiva and FlowJo Software.

### Flow Cytometric Analysis

Expression of B cell surface markers was performed by flow cytometry. 1*10^5^ cells (B cell lines or human primary B cells) were stained with anti-CD19-PE/Cyanine7 (clone HIB19, BioLegend), anti-CD20-Alexa647 (Rituximab, labeled with ThermoFisher AlexaFluor647 Labeling Kit), anti-FcγRIIb-Alexa647 (clone 2B6, labeled with ThermoFisher AlexaFluor647 Labeling Kit), anti-CD55-PerCP/Cyanine5.5 (clone JS11, BioLegend) or anti-CD59-PE (clone H19, BioLegend). Dead cells were excluded by subsequent staining with DAPI. Samples were acquired on a FACSCantoII (BD Biosciences) and analyzed using FACSDiva and FlowJo Software.

### C1q Binding ELISA

Interaction of CD20-specific mAbs with C1q was analyzed by enzyme-linked immunosorbent assay (ELISA). All incubation steps were performed at room temperature for 1 h. 100 µg/ml of mAbs were coated in 50 mM sodium bicarbonate buffer pH 9.6 (Sigma). After three washing steps with PBS 200 µl of blocking buffer (PBS containing 3% bovine serum albumin and 0.05% Tween-20) were added. After removal of blocking buffer increasing concentrations of native human C1q (Serotec) diluted in blocking buffer were added followed by three more washing steps. HRP-conjugated sheep anti human complement C1q (Serotec) was diluted 1:500 in blocking buffer. Plates were washed three times with PBS and TMB solution (Invitrogen) was added to detect anti-C1q-HRP. The reaction was stopped with 6% orthophosphoric acid.

### Quantification of GM_1_ Levels

Human B cells (Ramos, Daudi, P493.6, LCL1.11) or human peripheral blood leukocytes were incubated with AlexaFluor555-labeled cholera toxin subunit B (CT-B; ThermoFisher Scientific) diluted in RPMI 1640 medium for 10 min at 4°C. Bound CT-B was crosslinked with anti-cholera toxin subunit B antibody (anti-CT-B, rabbit serum; ThermoFisher Scientific) for 15 min at 4°C and GM_1_ levels were detected by flow cytometry on a FACSCytoFLEX S (Beckman Coulter Life Sciences). Human peripheral blood leukocytes were purified by RBC lysis and stained with anti-CD19-PE/Cyanine7 (clone HIB19; BioLegend) to detect GM_1_ levels on primary B cells. Data was analyzed with Flow Cytometry Analysis Software (FlowJo).

### Statistics

GraphPad Prism v8.3 was used for statistical analysis. Initially, shapiro-wilk test was used to determine Gaussian distribution of data sets. Subsequently, Kruskal-Wallis and Dunn´s multiple comparisons test (non-Gaussian distribution) or analysis of variance (ANOVA) followed by Sidak´s multiple comparisons *post hoc* test (Gaussian distribution) were performed. Alternatively, two-way RM ANOVA and multiple comparisons tests were applied. A detailed description of statistical tests used for individual experiments can be found in the respective figure legends.

## Results

**Figure 1:**
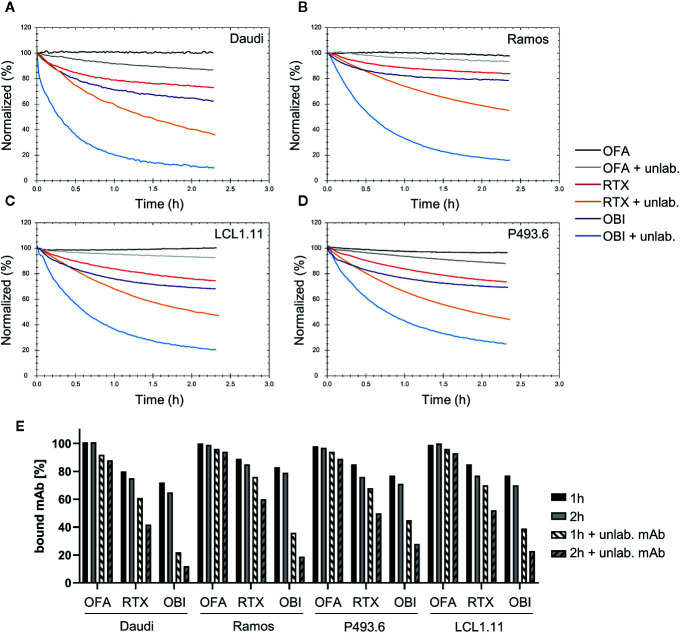
Binding stability of CD20 mAbs to human lymphoma B cell lines. Binding of 60 nM Ofatumumab (OFA), Rituximab (RTX), or Obinutuzumab (OBI) to Daudi **(A)**, Ramos **(B)**, LCL1.11 **(C)**, or P493.6 **(D)** cells was recorded until equilibrium was approached (not shown) followed by measurement of mAb dissociation either in plain cell culture medium or in presence of 60 nM of the respective unlabeled (unlab.) antibody. For all cell lines the dissociation of OFA was measured in presence of 180 nM unlabeled OFA (instead of 60 nM) to enhance possible cell-line differences. **(E)** Signal intensities were normalized to 100% at the beginning of the dissociation. The remaining signal after 1 h (black) and 2 h (gray) dissociation both in plain media (solid) and in presence of unlabeled antibody (shaded) are plotted for human B cell lines.

**Figure 2:**
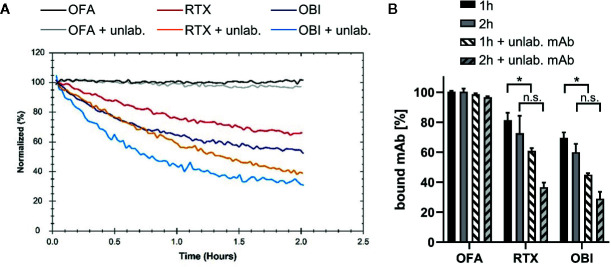
Binding stability of CD20 mAbs to human primary B cells. Binding of 60 nM Ofatumumab (OFA), Rituximab (RTX) or Obinutuzumab (OBI) to primary human B cells isolated from blood of healthy donors was recorded until equilibrium was approached (not shown) and then mAb dissociation was measured either in plain cell culture medium or in presence of 60 nM of the respective unlabeled antibody. **(A)** One out of three independent measurements shown. **(B)** Signal intensities were normalized to 100% at the beginning of the dissociation. The remaining signal after 1 h (black) and 2 h (gray) dissociation both in plain media (solid) and in presence of unlabeled antibody (shaded) are plotted. Bars show mean ± standard deviation of n=3 experiments using cells from different donors. For statistical analysis of signal intensities in plain media and in presence of unlabeled antibody for individual time points, two-way ANOVA and Tukey´s multiple comparison test were applied. *p<0.05, n.s. not significant.

A variety of B cell lines such as Raji (11, 23, 38, 43, 44), Ramos (44, 45), or Daudi (11, 38, 46) are routinely used to study cytotoxic IgG activity directed against CD20. When studying the interaction of CD20-specific mAbs, we previously found that binding to the Burkitt lymphoma cell line Daudi (47) is most stable for OFA, followed by RTX and least stable for OBI, due to the mAbs engaging in bivalent target binding to differing degrees (41). To extend our analyses, we evaluated the binding pattern of the three mAbs by real-time interaction analysis on additional human lymphoma B cell lines (the Burkitt lymphoma cell line Ramos (48) and the lymphoblastoid cell lines P493.6 (49) and LCL1.11) (**Figures 1A–D**), as well as primary human B cells (**Figure 2A**) purified from human peripheral blood (**Supplementary Figure 2**). Consistent with our previous data, OFA binding is most stable, followed by RTX, whereas OBI displayed the least stable binding (**Figures 1E** and **2B**). Also, in line with previous results, the number of cell-bound OFA molecules was barely influenced by the presence of free mAb in solution, whereas the number of cell-bound RTX and OBI molecules was clearly decreased by the presence of unbound mAb during the dissociation phase, implying more dynamic interactions, both on cell lines and on primary human B cells. Compared to RTX, the stability of bound OBI is even more strongly affected by the presence of free antibody in solution on all tested cell lines (**Figure 1E**), however this difference was less pronounced on primary human B cells (**Figure 2B**).

**Figure 3:**
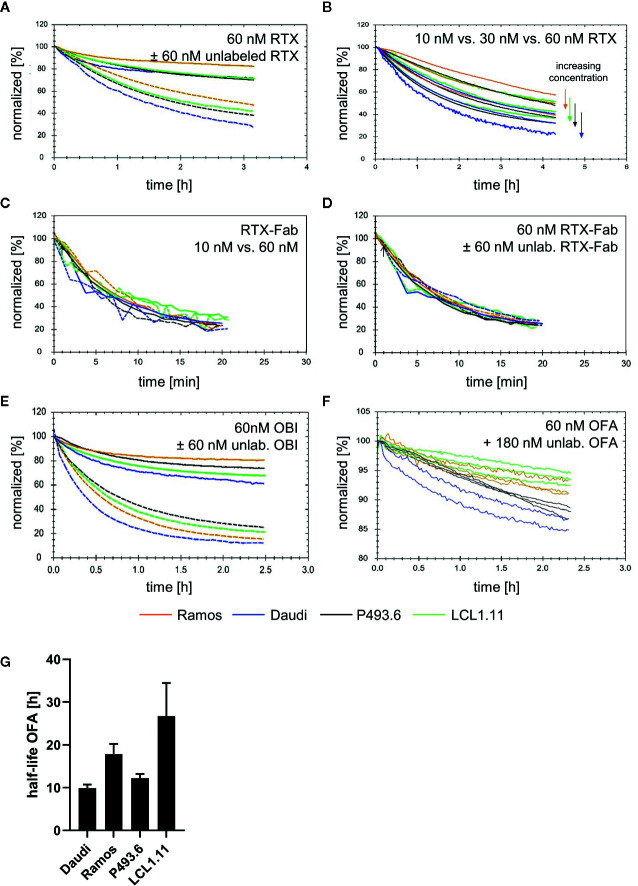
Binding stability of CD20 mAbs across different lymphoma B cell lines. **(A)** Dissociation after incubation with 60 nM fluorescein isothiocyanate Rituximab (FITC-RTX) in either plain medium (solid lines) or in the presence of 60 nM unlabeled RTX (dashed lines). **(B)** Dissociation after incubation with 10 nM, 30 nM or 60 nM FITC-RTX in the presence of equimolar amounts of unlabeled RTX. **(C)** Dissociation in plain medium after incubation with either 10 nM (dashed lines) or 60 nM (solid lines) FITC-RTX-Fab. **(D)** Dissociation after incubation with 60 nM FITC-RTX-Fab in either plain medium (solid lines) or presence of 60 nM unlabeled RTX-Fab (dashed lines). **(E)** Dissociation after incubation with 60 nM FITC-Obinutuzumab (OBI) in either plain medium (solid lines) or in the presence of 60 nM unlabeled OBI (dashed lines). **(F)** Dissociation after incubation with 60 nM FITC-Ofatumumab (OFA) in the presence of 180 nM unlabeled OFA. Note the different y-axis scaling. **(G)** Average half-life for bound OFA calculated from data shown in **(F)**. Bars represent mean ± standard deviation of n=3–4 independent measurements.

While confirming that the overall binding pattern of the CD20 mAbs is consistent across the tested cell lines as well as on primary human B cells, we also noticed differences in binding stability between the cell lines. On Ramos cells, RTX displayed most stable binding whereas the other three cell lines showed very similar dissociation patterns in plain cell culture medium (**Figure 3A**, solid lines). In the presence of free antibody in solution, the apparent off-rate for RTX was slowest for the dissociation from Ramos cells, followed by LCL1.11 cells, then P493.6 cells and lastly Daudi cells with the fastest apparent off-rate (**Figure 3A**, dashed lines, **Supplementary Table 1**). The stability of bound RTX further decreased with increasing concentrations for all cell lines (**Figure 3B**), with the half-life of bound RTX in the presence of free antibody in solution at 60 nM being roughly half compared to the half-life at 10 nM across all tested cell lines (**Table 1**). In contrast, the dissociation of RTX-Fab was neither influenced by the Fab concentration, nor by the presence of free ligand in solution (**Figures 3C, D**), which is a good indication for the interaction following a 1:1 behavior. Moreover, the stability of bound RTX-Fab did not significantly differ between the cell lines (**Figure 3D** and **Supplementary Table 2**), indicating that the differences in binding stability for RTX-IgG are due to secondary stabilizing effects, such as e.g. bivalency or Fc-interactions.

**Table 1:** Rituximab (RTX) binding stability at 10 nM and 60 nM was studied in the presence of unlabeled mAb in solution.

Cell line	RTX conc	k_d_ (1/s)	Half-life (min)	Fold change half-life
Ramos	10 nM	3.32E-05	348	
	60 nM	7.54E-05	153	0.44
Daudi	10 nM	7.17E-05	161	
	60 nM	1.28E-04	90	0.56
P493.6	10 nM	4.97E-05	232	
	60 nM	1.03E-04	112	0.48
LCL1.11	10 nM	5.14E-05	225	
	60 nM	9.69E-05	119	0.53

The first 2 h of dissociation were used to calculate dissociation rate constants and corresponding half-lives.

For OBI, the apparent off-rate as measured in plain medium was slowest for the dissociation from Ramos cells, followed by P493.6, LCL1.11 and lastly Daudi cells with the fastest apparent off-rate (**Figure 3E**, solid lines). This order changed when the stability of cell-bound OBI was studied in the presence of unlabeled antibody in solution: after 2 h the highest percentage of remaining OBI molecules was on P493.6 cells, followed by LCL1.11, Ramos and lastly Daudi cells (**Figure 3E**, dashed lines). In agreement with previously obtained data (41), the binding stability of OFA was not significantly influenced by the presence of equimolar amounts of free antibody in the 10-60 nM range (data not shown). Therefore, the stability of OFA was tested with 60 nM labeled antibody during the association, followed by three-fold molar excess of unlabeled antibody during the dissociation phase. OFA dissociated slowest from LCL1.11 cells, followed by Ramos and P493.6 cells and lastly Daudi cells with the fastest apparent off-rate (**Figures 3F, G**). Fitting a single exponential decay to the dissociation phase resulted in average half-lives of 26.7 h for LCL1.11, 17.8 h for Ramos, 12.2 h for P493.6 and 9.9 h for Daudi cells. For all three mAbs, both in absence and presence of unlabeled antibody, the apparent off-rate was fastest from Daudi cells.

**Figure 4:**
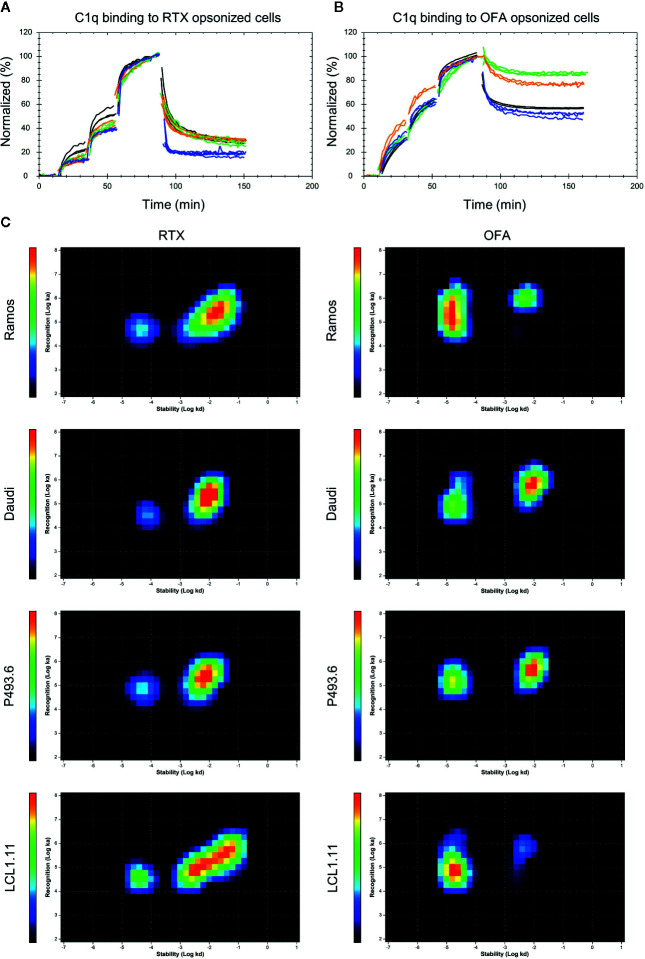
Binding of C1q to antibody opsonized B cells. Binding of fluorescent C1q to mAb opsonized Ramos (orange), Daudi (blue), P493.6 (black) and LCL1.11 cells (green) at concentrations of 1.4 nM, 3.9 nM, and 9.6 nM followed by dissociation. Cells were pre-incubated with either 60 nM unlabeled Rituximab (RTX) **(A)** or Ofatumumab (OFA) **(B)** for 1 h at room temperature prior to C1q binding and the antibody concentration was kept constant during the entire experiment. **(C)** InteractionMap for C1q binding to mAb opsonized cells. InteractionMap (IM) is an on/off-plot with each pixel representing a unique k_a_/k_d_ combination and heat-map coloration indicating how much each combination is contributing to the interaction analyzed. Per cell line n=3–4 replicates were calculated into one IM.

The off-rate, which is a measure for the binding stability, as well as bivalent target engagement of mAbs have been discussed as parameters that might influence the effectiveness of complement activation. We therefore set-up an assay to monitor C1q binding in real-time on living cells opsonized with CD20 antibodies to establish the kinetics of the C1q interaction. Binding to cells coated with either RTX or OFA clearly deviated from a 1:1 interaction which can be directly seen from the dissociation phase of the binding curves where a fraction of C1q quickly releases from the opsonized cells, whereas another fraction of C1q is more stably bound (**Figures 4A, B**). Data was evaluated with InteractionMap that depicts the number of 1:1-like interactions contributing to the overall binding pattern in an on-off plot. This type of analysis resulted in discovery of two distinct interaction components for all cell lines. These components primarily differed in their off-rates, which reflects the binding stability. The interaction of C1q to cells coated with OFA (**Figure 4B**) resulted in an over-all stronger and more stable interaction compared to cells coated with RTX (**Figure 4A**), whereas C1q binding to cells coated with OBI was too weak to give a clear signal above background (**Supplementary Figure 3**). Of note, C1q binding to OFA was not significantly superior to RTX or OBI in a cell-free enzyme-linked immunosorbent assay (ELISA), emphasizing the importance of a cellular model system for complex binding studies (**Supplementary Figure 4**).

Interaction analysis of C1q with Rituximab opsonized Daudi cells revealed affinity values in the range of 0.8-2.3 nM for the strong interaction and 32-70 nM for the weak interaction depending on the cell line (**Table 2**). The k_d_ value differed by more than 100-fold between the two interactions, resulting in half-lives above 2 h for the stable interaction (**Figure 4C**, peaks toward the left on the InteractionMaps) and 1-2 min for the transient interaction (**Figure 4C**, peaks toward the right on the InteractionMaps). For all cell-lines the strong interaction component contributed less to overall C1q binding, with an estimated strong binding site fraction of 0.13 for Daudi, 0.15 for Ramos 0.17 for LCL1.11 and 0.20 for P493.6 cells at the tested C1q concentrations (**Figure 4C**). The half-life for strongly bound C1q molecules was longest on LCL1.11 cells, followed by Ramos and P493.6 cells, whereas C1q displayed the shortest half-life on RTX-opsonized Daudi cells. Taking both half-life and fraction of binding sites into account, C1q binding was clearly least stable on Daudi cells, which corresponds with RTX also displaying least stable binding to these cells.

**Table 2:** Kinetic values for C1q interaction components on mAb opsonized cells as extracted by global InteractionMap analysis for each condition.

Strong interaction component					
	k_a1_ (1/(M*s))	k_d1_ (1/s)	K_D1_ (M)	half-life (min)	fraction binding sites
RTX-Ramos	5.2E+04	4.8E-05	9.3E-10	240	0.15
RTX-Daudi	3.4E+04	7.8E-05	2.3E-09	147	0.13
RTX-P493.6	6.7E+04	5.6E-05	8.3E-10	208	0.20
RTX-LCL1.11	3.4E+04	4.0E-05	1.2E-09	286	0.17
OFA-Ramos	2.3E+05	1.7E-05	7.1E-11	694	0.77
OFA-Daudi	1.1E+05	2.1E-05	1.9E-10	558	0.42
OFA-P493.6	1.5E+05	1.7E-05	1.1E-10	700	0.43
OFA-LCL1.11	7.6E+04	1.8E-05	2.4E-10	649	0.85
**Weak interaction component**					
	k_a2_ (1/(M*s))	k_d2_ (1/s)	K_D2_ (M)	half-life (min)	fraction binding sites
RTX-Ramos	2.0E+05	1.4E-02	7.0E-08	1	0.85
RTX-Daudi	1.8E+05	9.1E-03	4.9E-08	1	0.87
RTX-P493.6	2.1E+05	6.7E-03	3.2E-08	2	0.80
RTX-LCL1.11	1.5E+05	9.8E-03	6.4E-08	1	0.83
OFA-Ramos	1.0E+06	5.3E-03	5.1E-09	2	0.23
OFA-Daudi	6.1E+05	8.8E-03	1.4E-08	1	0.58
OFA-P493.6	4.5E+05	8.8E-03	2.0E-08	1	0.57
OFA-LCL1.11	5.7E+05	4.3E-03	7.6E-09	3	0.15

Depending on the cell line, the interaction with OFA opsonized cells resulted in affinities of 0.07-0.2 nM for the stable component and 5-20 nM for the weaker binding component. Half-lives for the weak C1q binding component were 1-3 min on OFA opsonized cells and thus very similar to those observed for the weak binding component on RTX opsonized cells. Half-lives for the strong C1q binding component were longer for OFA opsonized cells than for RTX opsonized cells. Concerning the cell-lines incubated with OFA, the half-life for strongly bound C1q was shortest on Daudi cells (9.3 h), followed by LCL1.11 cells (10.8 h) and very similar on Ramos and P493.6 cells (both 11.6 h) (**Table 2**). The fraction of strong C1q binding sites was similar on OFA-opsonized Daudi and P493.6 cells with 0.42 and 0.43 respectively, whereas it was higher with 0.77 for OFA-opsonized Ramos and highest on LCL1.11 cells with 0.85, which correlates with the OFA binding stability on these cell lines. Overall differences in C1q binding strength were more pronounced when comparing RTX and OFA than the differences between the cell lines.

**Figure 5:**
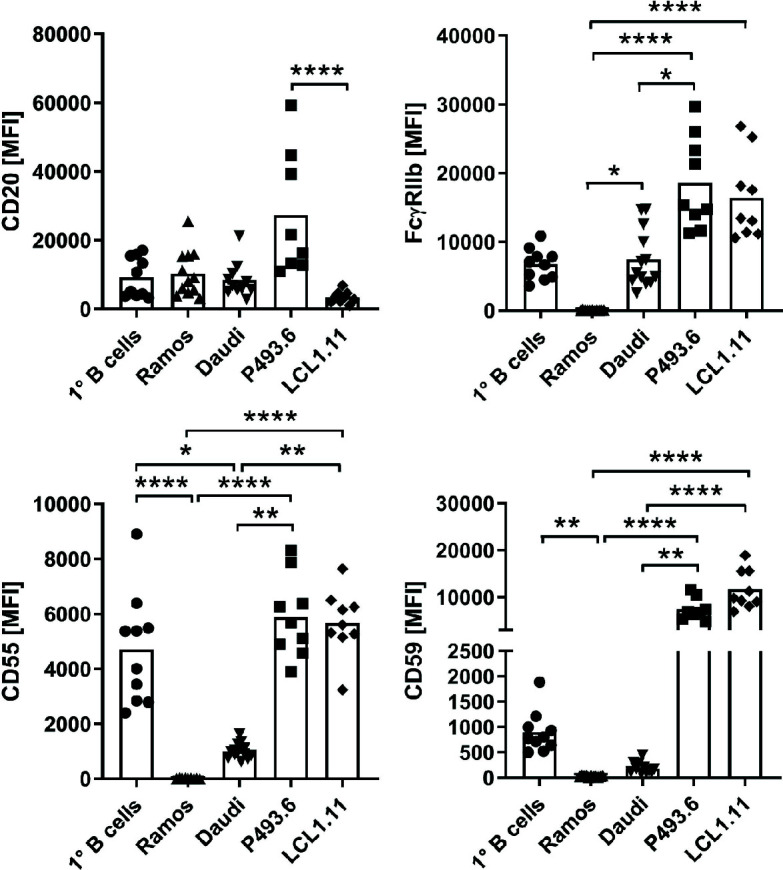
Comparison of surface marker expression. Expression of CD20, inhibitory FcγRIIb and complement-regulatory proteins CD55 and CD59 was assessed by flow cytometry on various B cell lines in comparison to primary human peripheral blood B cells from healthy donors. Symbols indicate biological replicates of the median fluorescence intensity (MFI) of the respective marker. Bars show statistical mean. Kruskal-Wallis test and Dunn´s multiple comparisons *post-hoc* test were applied to calculate statistical significance. *p<0.05, **p<0.01, ****p<0.0001.

Next, we analyzed the expression of selected cell surface proteins that might influence antibody binding stability and complement activation on these cell lines, as well as on primary human B cells to see if these could explain the observed differences (**Figure 5**). While Ramos and Daudi cells expressed CD20 to comparable degrees as primary B cells, CD20 expression on LCL1.11 cells was very low. In contrast to the other cell lines, P493.6 cells showed variable but elevated expression levels of CD20 and additionally high levels of inhibitory FcγRIIb that can potentially interact with the Fc portion of the antibodies (28). Daudi cells expressed FcγRIIb to comparable levels as primary B cells, whereas expression was higher on LCL1.11 cells and absent on Ramos cells. Consequently, a direct correlation for expression levels of CD20 and FcγRIIb did not become apparent in regards to antibody binding stability and subsequent C1q capture. The expression of the complement regulatory proteins CD55, an inhibitor of C3 and C5 convertases on the cell surface (50), and CD59 which blocks MAC formation (51) was also analyzed as these might have an impact on how C1q capture translates to complement activation. We found that CD55 expression was highest on P493.6 cells, LCL1.11 and primary B cells. CD59 expression was elevated on P493.6 and LCL1.11 cells in comparison to primary B cells. In contrast, Daudi and Ramos cells expressed only low levels or even completely lacked CD55 and CD59 confirming previous reports (52).

**Figure 6:**
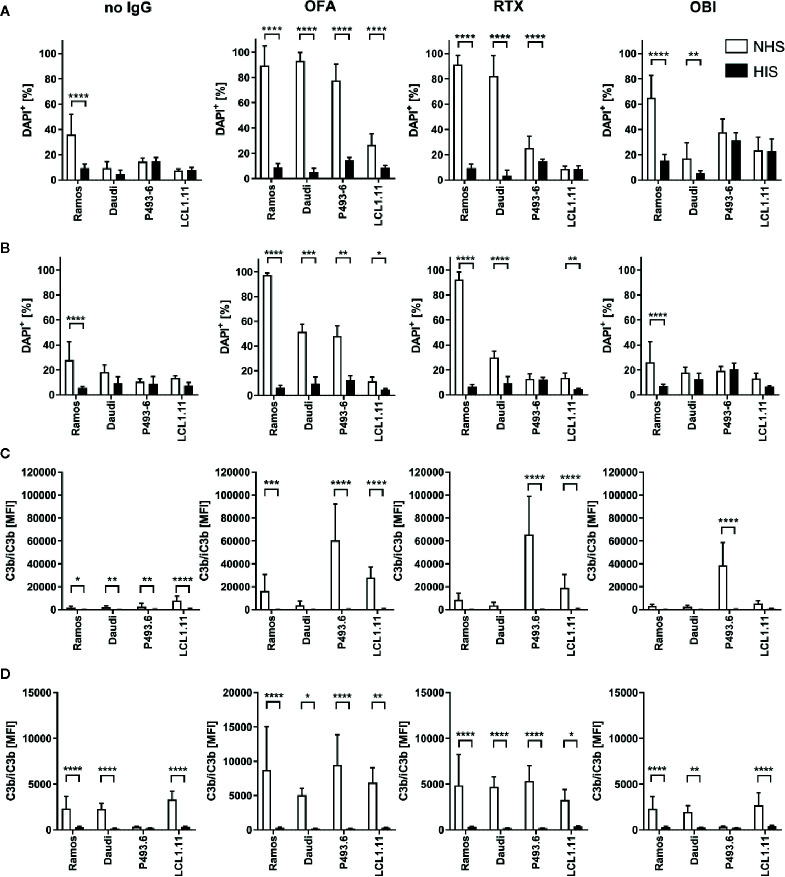
Complement dependent lysis and C3b deposition on B cell lines. Ramos, Daudi, P493.6 and LCL1.11 B cell lines were treated with 20 µg/ml **(A, C)** or 2 µg/ml **(B, D)** anti-CD20 mAb and 20% human serum (white bars) for 30 min at 37°C. As controls, cells were treated with heat-inactivated serum (black bars) or with serum in absence of anti-CD20 mAb. **(A, B)** Dead cells were quantified by flow cytometry analysis of DAPI stained cells. **(C, D)** Quantification of C3b/iC3b deposition on B cells. Bars show statistical mean ± standard deviation of n=3–5 independent experiments each using three to seven human serum samples **(A, C)** or n=2–3 independent experiments each using three to four human serum samples **(B, D)** per cell line. For statistical analysis, two-way ANOVA and Tukey´s multiple comparison test were applied. *p<0.05, **p<0.01, ***p<0.001, ****p<0.0001.

**Figure 7:**
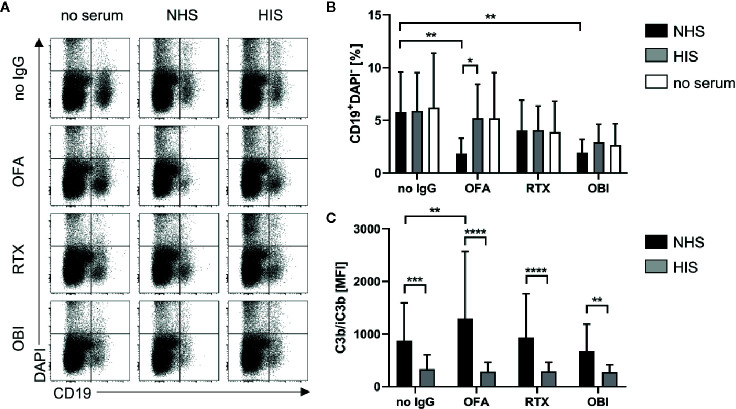
Complement dependent lysis and C3b deposition on primary human B cells. CDC and C3b deposition were compared for primary human B cells derived from healthy donor blood upon treatment with 20 µg/ml Rituximab (RTX), Ofatumumab (OFA), or Obinutuzumab (OBI) in absence (white) or presence of 20% human serum (NHS, black bars) or heat-inactivated serum (HIS, gray bars) for 30 min at 37°C. **(A)** Exemplary dot plots of CD19 and DAPI staining. **(B)** Quantification of living CD19^+^DAPI^-^ cells. **(C)** Quantification of C3b/iC3b deposition on B cells. Bars show mean and standard deviation of n=4 independent experiments using PBMCs from different donors and treated with four human sera each. For statistical analysis, two-way ANOVA and Sidak´s multiple comparison test were applied. *p<0.05, **p<0.01, ***p<0.001, ****p<0.0001.

Given the remarkable differences observed for the lymphoma cell lines, we subsequently assessed the functional consequences of distinct CD20, CD55 and CD59 expression for induction of complement-dependent cell lysis (CDC) induced by the different CD20-specific IgG (**Figure 6**). Upon addition of surface-saturating doses of OFA (20µg/ml) normal human serum (NHS) was able to significantly induce cell death in all cell lines tested (**Figure 6A**, **Supplementary Figure 5**). At this dose, even OBI mediated CDC in Ramos and Daudi cells and RTX was in addition able to kill P493.6 cells. At lower antibody doses, CDC induction was overall decreased. Still, OFA retained its superior capacity to induce complement-dependent cell death, followed by RTX, while OBI was hardly able to induce CDC anymore (**Figure 6B**, **Supplementary Figure 6**). Of note, differences between the cell lines were not restricted to CDC induction. As OBI has previously been described to induce apoptosis (26) we also assessed the capacity of CD20-specific mAbs to induce direct killing of B cells. Indeed, following incubation of cells with anti-CD20 mAbs in absence of serum enhanced cell killing could only be observed for high-dose OBI treatment but was also restricted to P493.6 and LCL1.11 cells (**Supplementary Figures 7A, B**), the cell lines showing the highest or lowest CD20 expression levels, respectively. This suggests that other factors beyond CD20 expression are involved in this killing mechanism. In contrast to assays with human lymphoma cell lines where successful initiation of CDC resulted in an increase of DAPI^+^ cells, it was not possible to detect dead B cells in primary human samples. Instead, primary B cells seemed to disintegrate during the incubation time. We therefore quantified living, *i.e.* DAPI^-^, B cells instead and observed that only OFA was able to cause a significant reduction in the presence of human serum (**Figures 7A, B**). With respect to direct cell killing by the mAbs, a non-significant reduction in viable B cells could be observed upon OBI addition (**Supplementary Figure 7C**).

Induction of CDC is the ultimate result of complement activation. One crucial step during the activation cascade is, however, deposition of complement C3 on the cell surface. In addition to its role in advancing the activation cascade the cleavage product C3b also poses as a ligand for complement receptors expressed on phagocytic cells thereby marking target cells for complement-dependent phagocytosis (CDCP) (5). Given that CDCP might therefore also be involved in complement-dependent effector functions of cytotoxic IgG *in vivo*, we also investigated deposition of C3b (and its inactivated form iC3b) upon treatment of B cells with anti-CD20 IgG. C3b could only be observed upon addition of anti-CD20 IgG and normal human serum, but not heat-inactivated serum (**Figures 6C, D**, and **7C**), and C3b levels were highest on P493.6 cells but surprisingly low on Ramos, Daudi and LCL1.11 cells. Potentially, sufficient complement activation (including C3b deposition) triggers lysis of cells so that C3b on these cells can no longer be detected. Consequently, only cells that are not killed by the complement system would still show elevated C3b levels. This might also explain higher C3b detection upon treatment with the lower antibody dose (**Figure 6D**) and the minor C3b deposition on primary human B cells (**Figure 7C**). Even though complement activation in this scenario might be insufficient to trigger direct cell killing *via* MAC formation one cannot exclude that the deposited C3b would be able to induce phagocytosis and thus contribute to target cell killing.

**Figure 8:**
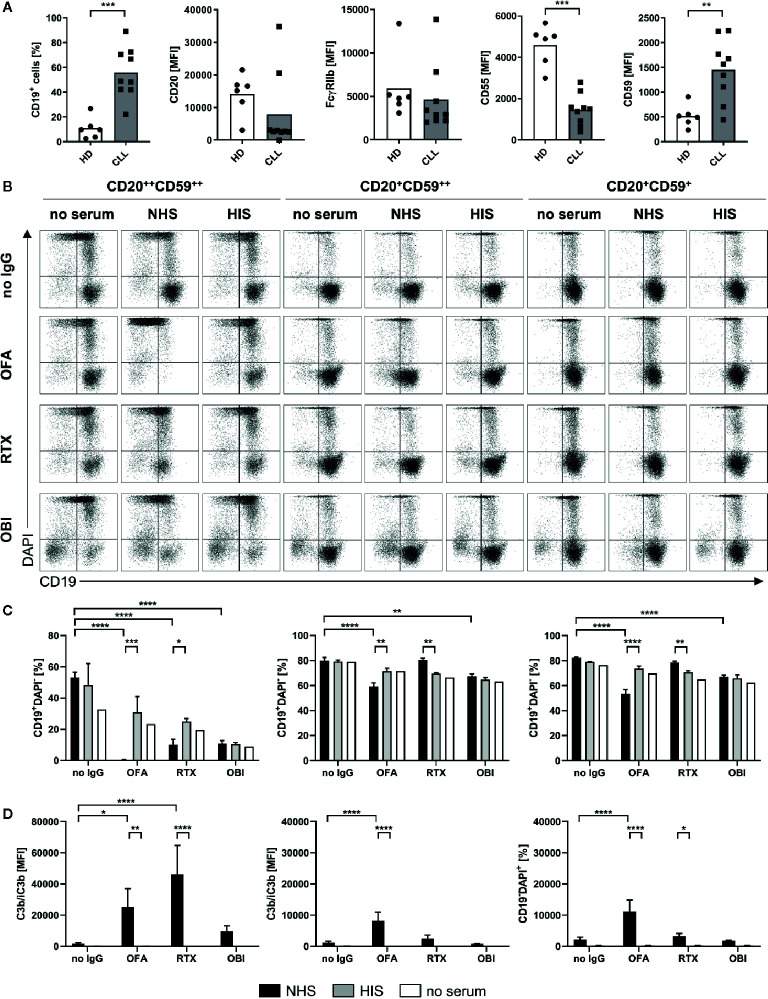
Complement dependent lysis and C3b deposition on primary human chronic lymphocytic leukemia (CLL) B cells. Complement-dependent cytotoxicity (CDC) and C3b deposition were compared for primary human B cells derived from CLL patients with distinct expression profiles of CD20 and CD59 upon treatment with 20 µg/ml Rituximab (RTX), Ofatumumab (OFA), or Obinutuzumab (OBI) in absence (white) or presence of 20% normal (NHS, black) or heat-inactivated (HIS, gray) human serum for 30 min at 37°C. **(A)** Flow cytometry expression analysis of CD19 and CD20, FcγRIIb, CD55, and CD59 on CD19^+^ cells of healthy (HD) or CLL B cells. **(B)** Exemplary dot plots of CD19 and DAPI staining. **(C)** Quantification of living CD19^+^DAPI^-^ cells for three to four sera. **(D)** Quantification of C3b/iC3b deposition on CD19^+^ cells. Bars show mean ± standard deviation of three to four serum samples. For statistical analysis, Mann Whitney test **(A)** or two-way ANOVA and Sidak´s multiple comparison test **(C, D)** were applied. *p<0.05, **p<0.01, ****p<0.0001.

CD20-specific antibodies are used to treat a variety of B cell malignancies, however for chronic lymphocytic leukemia (CLL) a reduced therapeutic potential has been observed (52). This was previously attributed to the fact that CLL B cells have been shown to express lower levels of CD20 (53–55) that might in turn reduce effector functions by anti-CD20 IgG, especially those dependent on complement activation (56). Given that we noticed CD20 expression levels to not be the sole factor determining efficacy of complement activation we compared B cells from 9 different CLL patients with respect to B cell surface marker expression (**Figure 8A**). As previously described, CD20 was expressed at lower levels on most CLL B cell samples however two of the donors did show elevated expression. In comparison to B cells from healthy donors, CLL B cells were characterized by homogenous decreased CD55 expression. In contrast, CD59 expression varied largely between donors but was overall increased. This suggests a reduced susceptibility toward MAC formation and therefore CDC but potentially an accumulation of active C3b on the B cell surface. Within the CLL patient cohort we identified three samples with different profiles regarding CD20 and CD59 that were further submitted to our CDC assay. Upon treatment with anti-CD20 IgG and human serum, CD20 turned out to be the more prominent determinant of complement-induced cytotoxicity as the strongest reduction of living B cells (CD19^+^DAPI^-^) by OFA was observed in the CD20^++^ sample. For this donor, RTX also caused a significant decrease in presence of NHS in comparison to HIS while no differences could be observed for OBI (**Figures 8B, C**). In addition, OFA and RTX, but not OBI, induced significant deposition of complement C3b (**Figure 8D**). Lower CD20 expression in the second and third CLL sample was associated with dampened OFA and abrogated RTX induced reduction of living B cells, irrespective of the CD59 expression level (**Figures 8B, C**). Surprisingly, we observed an increase in CD19^-^DAPI^-^ cells consistently upon treatment of CLL cells with OBI (**Figure 8B**, **Supplementary Figure 8**). Independent of cell killing this suggests that OBI might be able to induce CD19 loss on B cells as has previously only been described for RTX (57, 58). Accordingly, an increase of CD19^-^ cells could also be noticed upon RTX addition for the CD20^++^ CLL sample (**Figure 8B**, **Supplementary Figure 8**). Of note, CLL PBMC samples were stored frozen before the assay. To exclude an impact of freezing on susceptibility for CDC we also performed the assays with healthy human PBMCs following frozen storage (**Supplementary Figure 9**). Indeed, cells previously frozen were much more sensitive toward anti-CD20 mAb induced CDC as OFA, RTX and even OBI caused significant cell death and C3b deposition not observed with fresh PBMCs.

## Discussion

By applying real-time interaction analysis, we have previously shown that the stability of bound mAbs relates to the fraction of molecules that engage in bivalent binding. Accordingly, the highest fraction of bivalently bound antibody and the least dynamic binding pattern can be seen for OFA, while OBI displays the most dynamic pattern least stabilized by bivalency. Even though the present study confirmed these observations for additional B cell lines as well as primary B cells, we also observed that binding stability of anti-CD20 IgGs varies between human B cell lines. For instance, the stability of RTX in the presence of unlabeled mAb in solution was roughly two-fold less on Daudi compared to Ramos cells during the first hour of dissociation while primary human B cells displayed less variation with apparent off-rates for RTX being similar to those observed on Daudi cells. Differences between cell types were, however, smaller than differences between the antibodies, with e.g. OFA having a 10-fold longer binding half-life than RTX on Daudi cells.

These differences further translate to specific kinetics of C1q capture by antibody opsonized cells: Whereas the overall binding stability of C1q seems to correlate with OFA binding stability, C1q binding to RTX-opsonized cells was overall quite similar, except for RTX-opsonized Daudi cells that exhibited the weakest C1q capture and in line with this also least stable RTX binding. Binding of C1q to OFA opsonized cells was thus clearly stronger than for RTX on all tested cell lines, whereas binding of C1q to OBI opsonized cells could not be detected. Bivalent target engagement therefore positively correlates with strong C1q capture for the investigated anti-CD20 IgGs and expanding this analysis to a larger panel of anti-CD20 IgGs would be of interest, as contrasting observations have been made for other antigens (19, 37). The real-time binding assay presented in this study also allowed to resolve the presence of two interactions, as well as their kinetic and affinity values for C1q binding to anti-CD20 IgG opsonized cells. The two interactions differed mainly with respect to their dissociation rate constant k_d_, indicating that one fraction of C1q molecules is bound noticeably more stable than the remaining fraction. The interaction peaks of both C1q binding components as displayed in InteractionMaps were elongated in the y-axis direction, indicating heterogeneity in how C1q recognizes its binding partners, which is a sign of multivalent target engagement. As InteractionMap assumes that a binding curve can be explained by the weighted sum of individual 1:1 binding curves, multivalent binding becomes visible as a poorly defined k_a_ value that is seemingly changing during the interaction. This is due to the ratio of unbound receptors versus bound receptors decreasing faster than predicted as one ligand binds multiple targets, causing the rate of ligand-target complex formation to slow down more than what is expected according to a 1:1 model. This is especially noticeable for interaction peaks that represent a high percentage of binding sites such as the strong C1q binding component on OFA-opsonized Ramos cells. For interaction peaks that represent minor binding fractions this is less or not at all visible as the information contained in the binding traces is not sufficient to capture the exact shape of the interaction peak. Both InteractionMap peaks thus presumably represent multivalent binding of C1q to cell-bound IgG Fc-domains. The strong interaction component likely represents binding of C1q to IgG hexamers, whereas the less stable interaction component likely represents binding of C1q to IgG-Fc multimers that are smaller than hexamers, which have been shown to result in some CDC activity (24, 36). A general fast association for C1q is in agreement with previous reports and also explains the fast on-set of CDC, reaching maximum killing levels within 10 min even at C1q concentrations as low as 1 µg/ml (21, 38). The apparent affinity for C1q binding to OFA coated Daudi cells, as determined with an end-point assay, has been reported previously to be 16 nM (21, 38, 59), which is close to the value reported here for the less stable interaction component. Data resolution for end-point affinity measurements is typically not sufficient to discriminate between individual interaction components with different affinities. Of note, the C1q binding assay set-up in this study does not capture binding to IgG-Fc monomers, as binding is only detected if C1q concentrations are getting closer to the affinity value of the interaction, *i.e.*, around 10–100 µM for binding to Fc monomers (39) which might explain the lack of binding observed for OBI.

Regarding the functional consequences of C1q binding to mAb opsonized target B cells, we systematically compared complement mediated cell killing by RTX, OFA and OBI on human lymphoma cell lines and human primary B cells differing in expression levels of CD20 and complement regulatory proteins. Similar to what was previously reported (52), Ramos cells proved to be extremely sensitive in this assay as all three mAbs, and to a lesser extent serum in absence of anti-CD20 IgG, are able to significantly induce complement-dependent cell death. A potential explanation for Ramos cells being very sensitive to CDC is the strong C1q capture in combination with the absence of complement regulatory proteins. Even though LCL1.11 cells also display a strong C1q capture, CDC sensitivity is clearly reduced compared to Ramos cells, which might be explained by a lower expression of CD20 and the high expression of both CD55 and CD59. The low expression of both complement regulatory proteins might also account for the rather efficient CDC in Daudi cells, despite having the least strong C1q capture among the investigated cell lines. Surprisingly, P493.6 cells which strongly express CD20 and also nicely capture C1q were quite resistant to CDC induction supporting the notion that expression levels of complement regulatory proteins rather than CD20 determine the efficacy of complement-dependent killing of B cell lines. A decreased susceptibility for CDC and complement activation in general could also be observed for freshly isolated primary human B cells despite levels of CD20 and CD59 that are comparable to Ramos and Daudi. Elevated expression levels of CD55 on primary B cells might instead explain reduced complement activation. In fact, only OFA was able to consistently kill primary B cells as well as CLL B cells in a complement-dependent manner, again confirming the superior capacity of complement activation. In any case, the data presented here suggests that the choice of the *in vitro* model system and especially target B cell can dramatically impact conclusions about the extent of complement activation induced by CD20-specific mAbs. Given the contradictory literature (6, 56, 60–63), it therefore still remains to be seen whether CD20, complement regulator expression levels or a combination of both ultimately determine target cell susceptibility for complement dependent induction of cell death. Regarding the observed increase of CD19^-^ cells in CLL samples upon OBI (and to a lesser extent RTX) treatment, we speculate that OBI might induce a down-regulation of CD19 as has been previously described for RTX (57, 58). To the best of our knowledge this has not been observed before for OBI but data by Reddy et al. suggest that this might indeed be the case (29). However, the specifics of this potential reduction in CD19 would need to be investigated in more detail. One factor affecting our observations could be the short incubation time of only 30 min in this study which might enable capturing transitional effects during OBI induced cell killing.

There is accumulating evidence that efficiency of CDC induction by mAbs is dependent on a range of factors, such as the binding epitope and binding orientation of the mAb, as well as the antibody elbow hinge angle (30–33). This complexity is difficult to mimic when measuring C1q binding in artificial, isolated protein systems, especially since antigen mobility in the membrane might play a role (36). For anti-EGFR mAbs it has been shown that monovalent target engagement results in higher CDC efficacy. This could be a consequence of monovalent binding resulting in a higher number of IgGs bound and this, in turn, might increase the likelihood for the formation of multimeric IgG-Fc platforms suitable for C1q binding (37). For anti-CD20 mAbs, on the other hand, bivalent binding might enhance crosslinking of CD20 and thus lead to more efficient clustering of CD20 and bound mAbs. This notion is supported by recent structural studies showing that for RTX and OFA two Fabs belonging to different antibody molecules can interact with the same CD20 dimer. In contrast, only one Fab of the type II mAb OBI can be bound per CD20 dimer due to sterical constraints. As a consequence, it was suggested that type I mAbs can act as molecular seeds that promote the concatenation of IgG and CD20 molecules into larger molecular assemblies (25, 64). In line with this, it was previously observed that binding of type I anti-CD20 mAbs causes segregation of CD20 into detergent-resistant membrane domains which facilitates CDC induction (22). One could therefore speculate that the differences observed between the lymphoma cell lines and primary B cells could be caused by specific plasma membrane compositions resulting in differential distribution and mobility of CD20 and subsequently enhanced or decreased capture by anti-CD20 mAbs. Indeed, we identified low levels of sphingomyelin GM_1_, a typical component of organized membrane domains, in complement-resistant P493.6 cells while GM_1_ was highly present in the membrane of highly susceptible Ramos cells (**Supplementary Figure 10**). This suggests that Ramos cells have more organized microdomains with potentially pre-clustered CD20. Consequently, bivalent target engagement, IgG crosslinking and thereby the formation of suitable platforms for C1q capture may be facilitated. It should also be noted that GM_1_ levels in all tested cell lines were higher than in human primary cells.

One can further speculate that antibody induced clustering is more efficient in promoting IgG-Fc arrangements for C1q binding than increasing the total number of bound IgG that is not in clusters. This would explain why multivalent C1q binding to cells opsonized with the type I mAbs OFA and RTX, whose binding can act as molecular seeds and induces clustering of CD20, could be detected (22, 25). In contrast, no strong interaction of C1q was seen on cells opsonized with OBI, which binds CD20 dimers in a terminal conformation that does not allow the concatenation of several CD20 dimers into larger clusters (25). Differences in membrane mobility and mAb mediated clustering could also explain why type I anti-CD20 mAbs generally induce CDC more efficiently than anti-EGFR mAbs. Whether bivalent or monovalent target engagement is preferable for C1q binding might thus very well depend on the lateral receptor mobility in the cell membrane that may or may not be influenced by binding of the mAb. Interestingly, it has been reported that (Fab)_2_ fragments of type I anti-CD20 mAbs can activate complement in a C1q dependent manner (65). This suggests that other factors than IgG-Fc arrangement contribute to cell lysis by CDC for B cells, further strengthening the notion that complement activation is complex and conditions for optimal CDC activity may vary between target surface molecules.

## Data Availability Statement

The raw data supporting the conclusions of this article will be made available by the authors, without undue reservation.

## Ethics Statements

The studies involving human participants were reviewed and approved by Ethics Committee CAU Kiel Head: Christine Glinicke, Schwanenweg 20, D-24105 Kiel, Germany. Ethics Committee FAU Erlangen. Head: Kerstin Amann, Krankenhausstr. 12, D-91054 Erlangen, Germany. The patients/participants provided their written informed consent to participate in this study.

## Author Contributions

SB, AL, and FN designed experiments. SB, AL, AM, SK, and JL performed experiments. SB, AL, and SK analyzed data. MP provided essential reagents (CD20-specific mAbs, B cell lines, CLL samples). SB and AL wrote the manuscript. MP, FN, and JB revised the manuscripts. All authors critically reviewed the manuscript. All authors contributed to the article and approved the submitted version.

## Funding

This work was supported by the Deutsche Forschungsgemeinschaft (DFG) project grants DFG-TRR130-P13 and D-A-CH NI 711/9-1 to FN. FN was further supported by NIADS grant U01 AI-148119-01. AL was supported by Emerging Talents Initiative of the Friedrich-Alexander-University Erlangen-Nürnberg. AM was supported by Elitenetzwerk Bayern.

## Conflict of Interest

SB is an employee and JB is an employee and shareholder of Ridgeview Instruments AB.

The remaining authors declare that the research was conducted in the absence of any commercial or financial relationships that could be construed as a potential conflict of interest.
