# Supplementary material for: Complement-Dependent Activity of CD20-Specific IgG Correlates With Bivalent Antigen Binding and C1q Binding Strength
Source: Front Immunol. 2021 Jan 11;11:609941. doi: 10.3389/fimmu.2020.609941 (PMC7829346; doi:10.3389/fimmu.2020.609941)
Supplement: Supplementary file 1 [file Presentation_1.pdf]

## Supplementary Figure 1

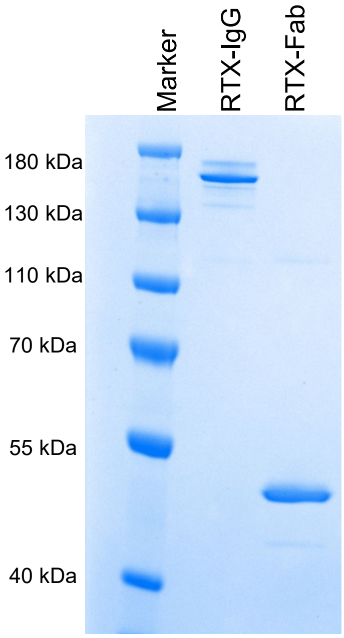


**Suppl Figure 1. Fab Fragmentation**

Non-reducing SDS PAGE analysis of full-lenght RTX and RTX-Fab that was generated via papain digestion.

# Supplementary Figure 2

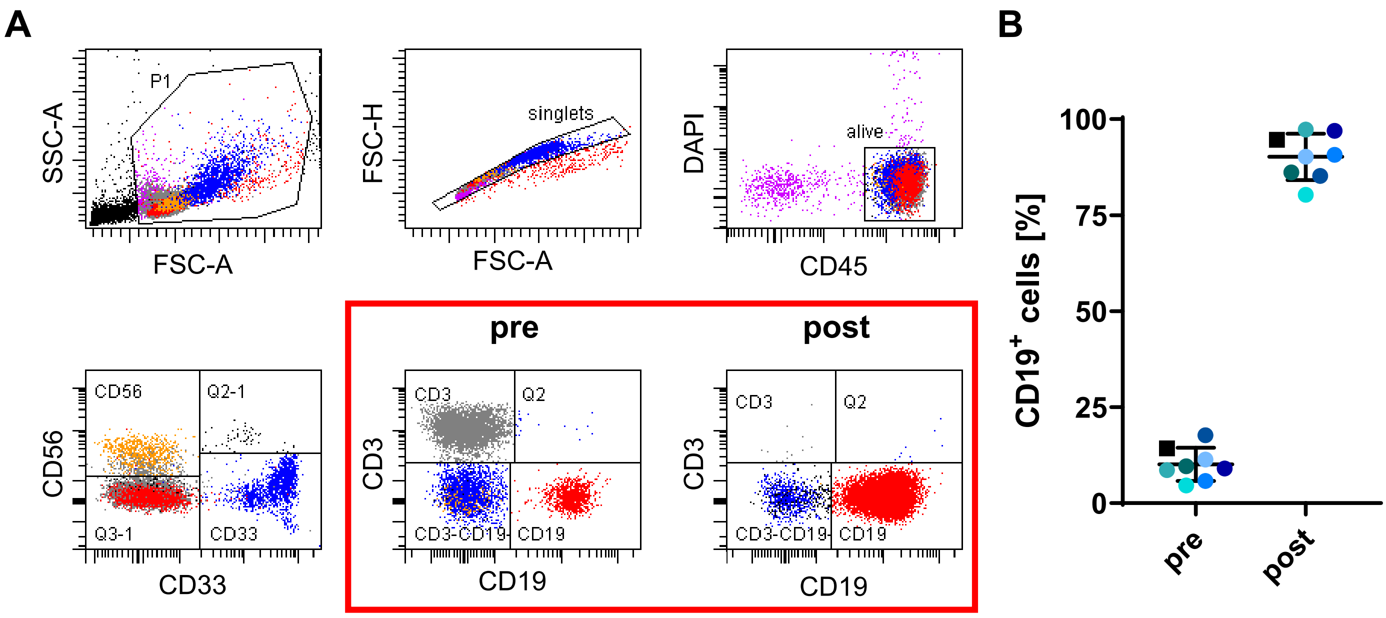


**Suppl. Figure 2. Quality control of human B cell isolation.**

Untouched primary human B cells were isolated from human peripheral blood by density gradient centrifugation and magnetic cell sorting. Purity of B cells (post) was analyzed by flow cytometry in comparison to PBMCs before isolation (pre) and reached 90 % on average. (A) Exemplary gating strategy starting with leukocytes and excluding duplettes. Cell lineages were assigned within living leukocytes ($CD45^+DAPI^-$) by surface marker expression: $CD56^+CD33^-$ NK cells, $CD33^+CD56^-$ monocytes, $CD3^+$ T cells, $CD19^+$ B cells. (B) Quantification of human B cell purity (% within $CD45^+DAPI^-$ cells) for n=8 different donors. Lines indicate statistical mean ± standard deviation.

# Supplementary Figure 3

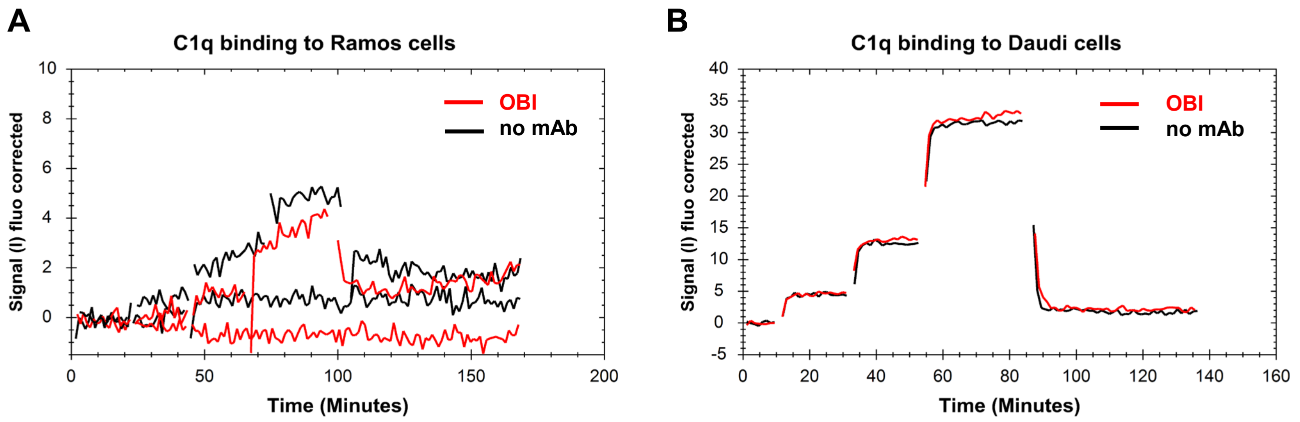


**Suppl Figure 3. C1q binding to OBI opsonized B-cells**

Binding of fluorescent C1q at concentrations of 1.4 nM, 3.9 nM and 9.6 nM to Ramos (A) or Daudi (B) cells followed by dissociation. For OBI opsonized cells (red), the unlabeled antibody was pre-incubated at 60 nM for 1 h at room temperature prior to C1q binding and the antibody concentration was kept constant during the entire experiment. As control, C1q binding to cells without any antibody was recorded (black).

## Supplementary Figure 4

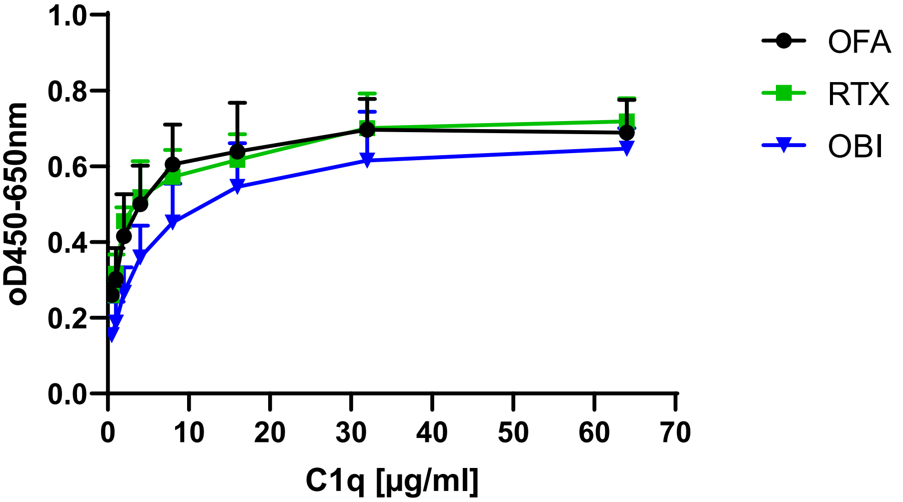


**Suppl. Figure 4. C1q binding to CD20-specific IgG.**

Binding of increasing amounts of recombinant human C1q to OFA, RTX and OBI was assessed by ELISA. Shown is the mean optical density (oD) at 450nm (background at 650nm substracted) of two independent experiments performed in technical duplicates.

## Supplementary Figure 5

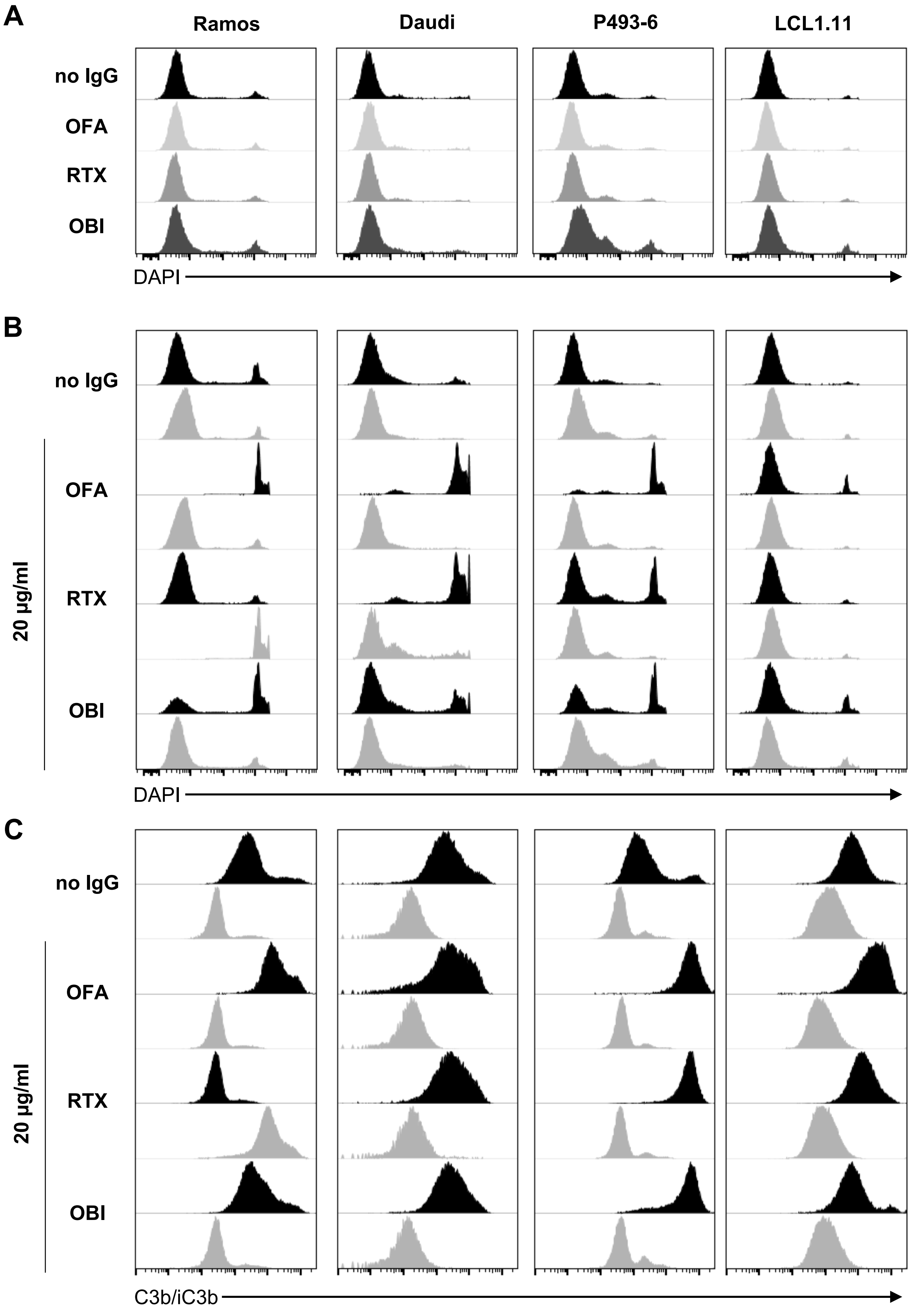


**Suppl Figure 5. Complement dependent lysis and C3b deposition on B cell lines.**

Exemplary histogram overlays of Ramos, Daudi, P493.6 and LCL1.11 lymphoma B cells upon treatment with **20 µg/ml** anti-CD20 IgG (RTX, OFA, OBI) and 20 % normal (NHS, black) or heat-inactivated (HIS, grey) human serum for 30 min at 37 °C. (A) DAPI⁺ cells following treatment with anti-CD20 IgG in absence of serum. (B) Flow cytometric analysis of DAPI⁺ cells following treatment with anti-CD20 IgG in prescence of NHS or HIS. (C) Flow cytometric analysis of C3b/iC3b deposition following treatment with anti-CD20 IgG in prescence of NHS or HIS. Shown is one exemplary experiment with serum from one donor each.

## Supplementary Figure 6

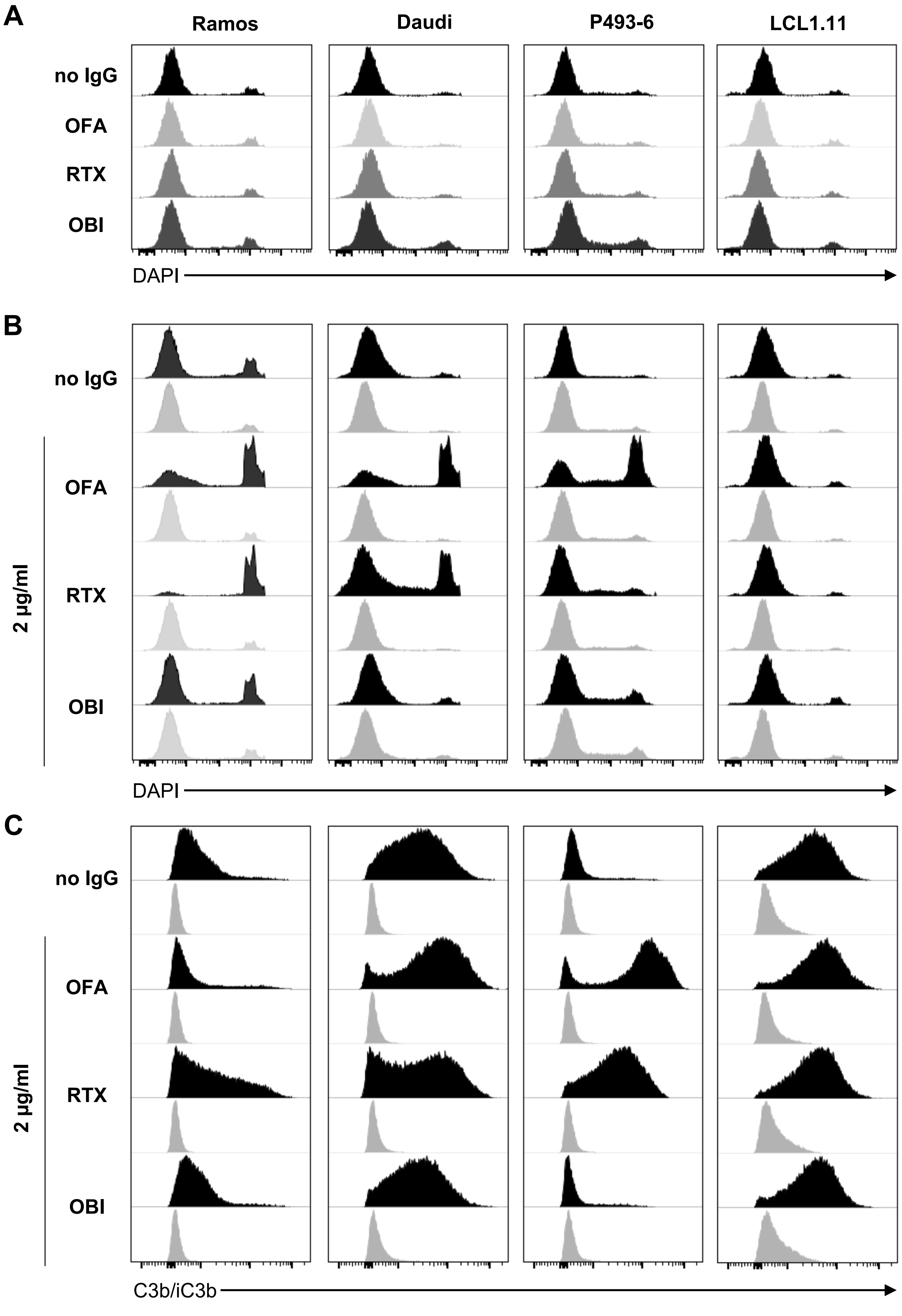


**Suppl Figure 6. Complement dependent lysis and C3b deposition on B cell lines.**

Exemplary histogram overlays of Ramos, Daudi, P493.6 and LCL1.11 B cells upon treatment with **2 µg/ml** anti-CD20 IgG (RTX, OFA, OBI) and 20 % normal (NHS, black) or heat-inactivated (HIS, grey) human serum for 30 min at 37 °C. (A) DAPI⁺ cells following treatment with anti-CD20 IgG in absence of serum. (B) Flow cytometric analysis of DAPI⁺ cells following treatment with anti-CD20 IgG in prescence of NHS or HIS. (C) Flow cytometric analysis of C3b/iC3b deposition following treatment with anti-CD20 IgG in prescence of NHS or HIS. Shown is one exemplary experiment with serum from one donor each.

# Supplementary Figure 7

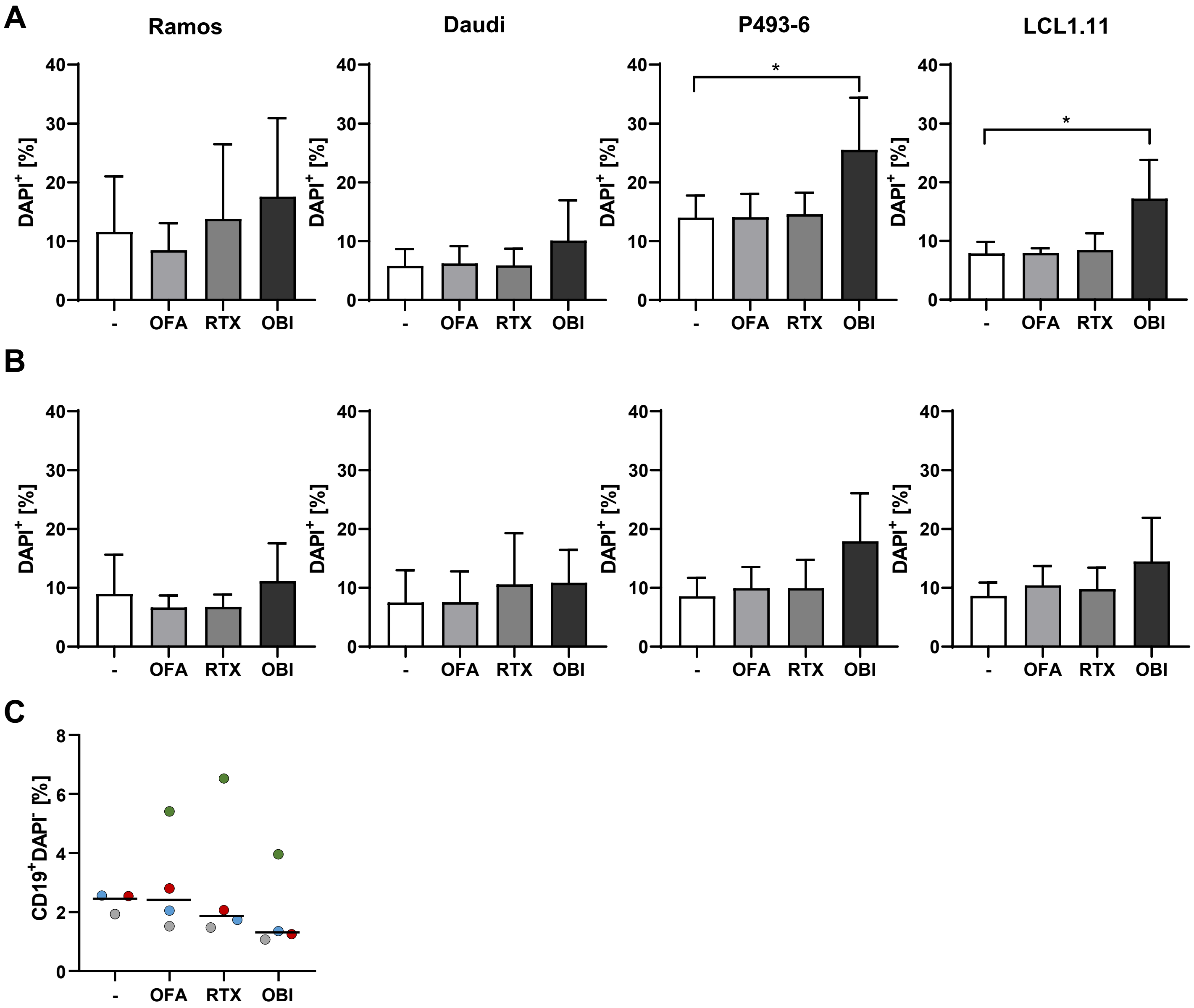


**Suppl. Figure 7. Direct cytotoxicity of therapeutic anti-CD20 IgG1 in absence of serum.**

Various B cell lines (A&B) and primary human B cells from healthy donors (C) were treated with 20 µg/ml anti-CD20 IgG1 (A) or 2 µg/ml (B) for 30 min at 37 °C to assess cytotoxic capacity of CD20-specific antibodies. Treated cells were stained with DAPI to label dead cells and analysed by flow cytometry. Bars show statistical mean ± standard deviation of 3-9 independent experiments. Data for primary human B cells represents 3-4 individual experiments using cells from different donors (depicted in individual colours). Horizontal line indicates statistical median. For statistical analysis Kruskal-Wallis test and Dunn´s multiple comparison test were applied. * $p < 0.05$

# Supplementary Figure 8

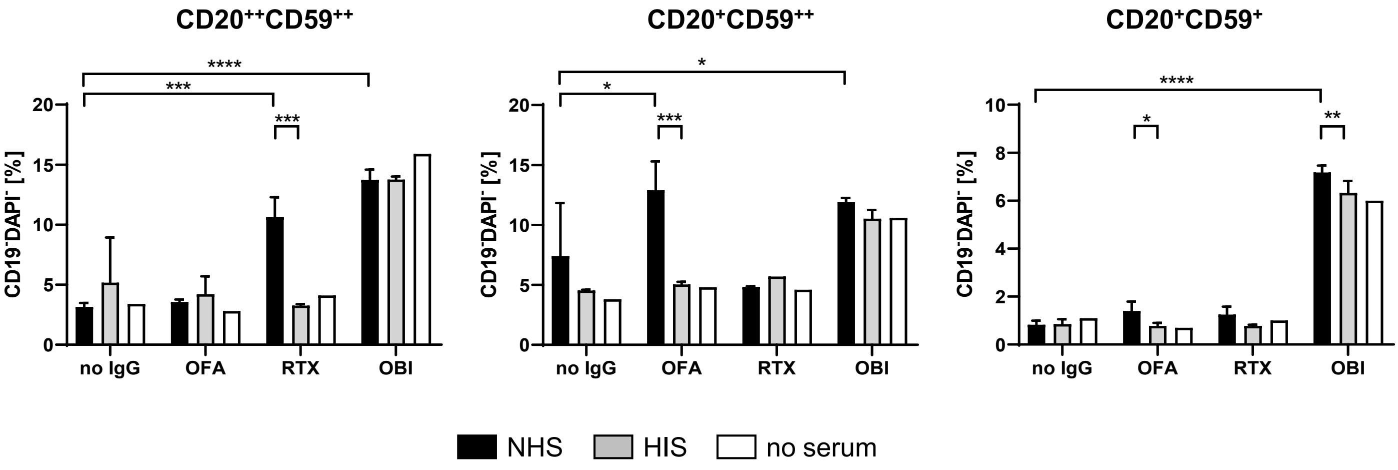


**Suppl Figure 8. Complement-dependent lysis of primary human CLL B cells.**

CDC was compared for primary human B cells derived from three CLL patients with distinct expression profiles of CD20 and CD59 upon treatment with 20 µg/ml RTX, OFA or OBI in absence (white) or presence of 20 % normal (NHS, black) or heat-inactivated (HIS, grey) human serum for 30 min at 37 °C. Quantification of $CD19^{-}DAPI^{-}$ cells for 3-4 sera. For statistical analysis, 2-way ANOVA and Sidak´s multiple comparison test were applied. *p<0.05, ** p<0.01, *** p<0.001, **** p<0.0001

## Supplementary Figure 9

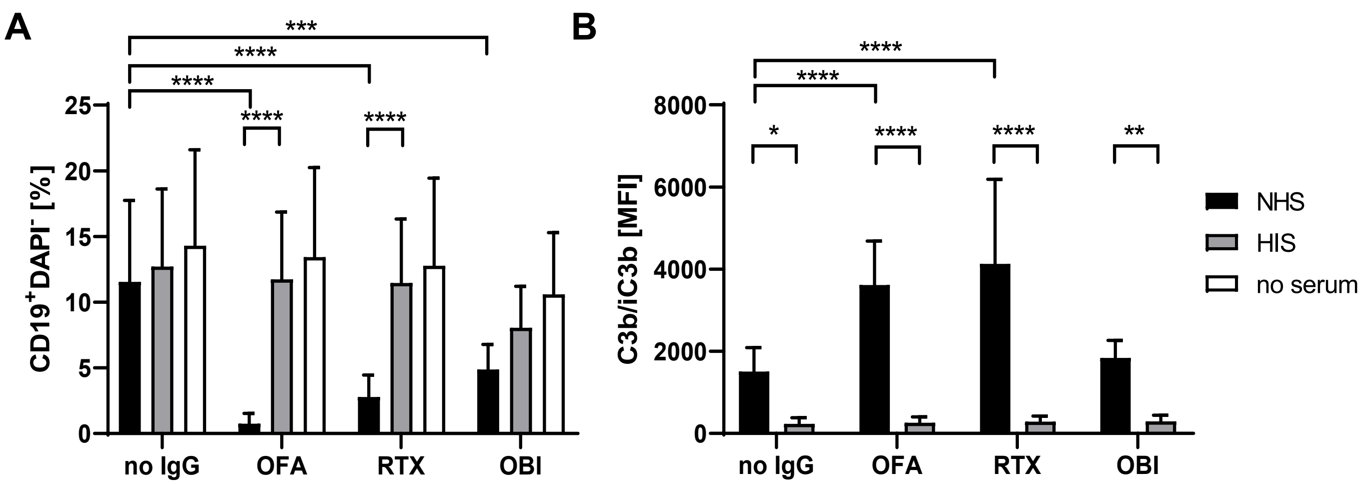


**Suppl Figure 9. Complement dependent lysis and C3b deposition on frozen, stored primary human B cells.**

CDC and C3b/iC3b deposition were compared for primary human B cells derived from healthy donors upon treatment with 20 µg/ml RTX, OFA or OBI in absence (white) or presence of 20 % normal (NHS, black) or heat-inactivated (HIS, grey) human serum for 30 min at 37 °C. In relation to CDC analysis of CLL B cells, PBMCs were stored at -80 °C before the experiment. (A) Quantification of living $CD19^{+}DAPI^{-}$ cells in n=4 independent experiments using different PBMC samples and 3-4 human sera each. (B) Quantification of C3b/iC3b deposition on $CD19^{+}$ cells. Bars show mean ± standard deviation of 14-16 serum samples. For statistical analysis, 2-way ANOVA and Sidakt´s multiple comparison test were applied. * $p<0.05$, ** $p<0.01$, *** $p<0.001$, **** $p<0.0001$

## Supplementary Figure 10

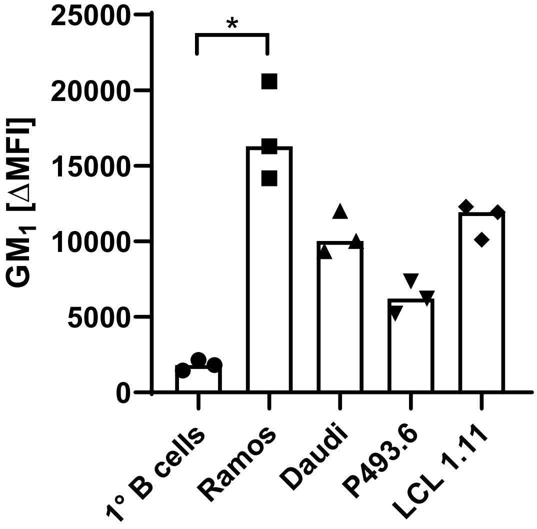


**Suppl Figure 10. Analysis of membrane composition of B cells.**

A) Fluorescent choleratoxin B was used to stain sphingomyelin $GM_1$, a marker for organized membrane domains, in the plasma membrane of primary human B cells and lymphoma cell lines Ramos, Daudi, P493.6 and LCL1.11 followed by flow cytometric quantification. Median fluorescence intensity (MFI) of unstained cells was substracted to obtain (ΔMFI). Bars indicate statistical mean of n=3 independent experiments. Kruskal-Wallis test and Dunn´s multiple comparisons post-hoc test were applied to calculate statistical significance. * $p<0.05$

# Supplementary Table 1

| | | Dissociation 0-1 h | | | Dissociation 1-2 h | | |
|---|---|---|---|---|---|---|---|
| Cells | antibody incubation | half-life (h) | % bound at 1 h | % additional dissociation in presence of unl mAb | half-life (h) | % bound at 2 h | % additional dissociation in presence of unl mAb |
| Daudi | FITC-RTX | 3.2 | 80 | | 12.3 | 75 | |
| | FITC-RTX + unl RTX | 1.3 | 61 | 23 | 2.0 | 42 | 44 |
| | FITC-OFA | 192.5 | 101 | | 183.4 | 101 | |
| | FITC-OFA + unl OFA | 8.4 | 92 | 9 | 26.4 | 88 | 12 |
| | FITC-OBI | 2.1 | 72 | | 6.7 | 65 | |
| | FITC-OBI + unl OBI | 0.4 | 22 | 70 | 1.2 | 12 | 81 |
| Ramos | FITC-RTX | 5.8 | 89 | | 14.4 | 85 | |
| | FITC-RTX + unl RTX | 2.2 | 76 | 15 | 2.9 | 60 | 30 |
| | FITC-OFA | 192.5 | 100 | | 102.4 | 99 | |
| | FITC-OFA + unl OFA | 16.2 | 96 | 4 | 23.4 | 94 | 5 |
| | FITC-OBI | 4.9 | 83 | | 28.0 | 79 | |
| | FITC-OBI + unl OBI | 0.6 | 36 | 57 | 1.0 | 19 | 77 |
| P493.6 | FITC-RTX | 3.7 | 85 | | 6.4 | 76 | |
| | FITC-RTX + unl RTX | 1.6 | 68 | 20 | 2.7 | 50 | 35 |
| | FITC-OFA | 25.5 | 98 | | 107.6 | 97 | |
| | FITC-OFA + unl OFA | 10.8 | 94 | 4 | 12.2 | 89 | 7 |
| | FITC-OBI | 3.0 | 77 | | 8.7 | 71 | |
| | FITC-OBI + unl OBI | 0.8 | 45 | 41 | 1.7 | 28 | 60 |
| LCL1.11 | FITC-RTX | 3.9 | 85 | | 6.6 | 77 | |
| | FITC-RTX + unl RTX | 1.7 | 70 | 17 | 2.3 | 52 | 32 |
| | FITC-OFA | 145.9 | 99 | | 192.5 | 100 | |
| | FITC-OFA + unl OFA | 26.3 | 96 | 3 | 26.5 | 93 | 6 |
| | FITC-OBI | 2.6 | 77 | | 6.9 | 70 | |
| | FITC-OBI + unl OBI | 0.7 | 39 | 49 | 1.4 | 23 | 67 |
| primary B cells donor 2 | FITC-RTX | 2.5 | 78 | | 4.3 | 66 | |
| | FITC-RTX + unl RTX | 1.3 | 62 | 20 | 1.6 | 40 | 39 |
| | FITC-OFA | 53.8 | 100 | | 192.5 | 101 | |
| | FITC-OFA + unl OFA | 19.6 | 99 | 2 | 60.4 | 97 | 4 |
| | FITC-OBI | 1.5 | 66 | | 3.9 | 55 | |
| | FITC-OBI + unl OBI | 0.8 | 46 | 31 | 2.2 | 33 | 39 |
| primary B cells donor 3 | FITC-RTX | 4.5 | 87 | | 34.2 | 86 | |
| | FITC-RTX + unl RTX | 1.2 | 62 | 28 | 1.4 | 36 | 58 |
| | FITC-OFA | 33.3 | 100 | | 34.0 | 98 | |
| | FITC-OFA + unl OFA | 192.5 | 99 | 1 | 12.6 | 96 | 3 |
| | FITC-OBI | 1.9 | 73 | | 7.1 | 66 | |
| | FITC-OBI+ unl OBI | 0.7 | 45 | 39 | 1.4 | 24 | 63 |
| primary B cells donor 4 | FITC-RTX | 2.6 | 79 | | 3.9 | 66 | |
| | FITC-RTX + unl RTX | 1.1 | 59 | 25 | 1.2 | 34 | 49 |
| | FITC-OFA | 192.5 | 101 | | 192.5 | 102 | |
| | FITC-OFA + unl OFA | 21.7 | 98 | 3 | 192.5 | 97 | 5 |
| | FITC-OBI | 1.8 | 70 | | 4.3 | 59 | |
| | FITC-OBI + unl OBI | 0.7 | 44 | 37 | 1.7 | 30 | 49 |

**Suppl. Table 1. Analysis of RTX binding stability across different B-cells.**

% of remaining signal as well as half-lives calculated from the dissociation rate constants are given for the the first and second hour during dissociation. The additional dissocation in presence of unlabeled (unl) mAb was calculated as 1- (% bound in presence of unl mAb/% bound in absence on unlabeled mAb).

# Supplementary Table 2

| Cell line & RTX-Fab conc. | $k_d$ (1/s) | half-life (min) |
|---|---|---|
| Daudi 10 nM | 1.55E-03 | 7.5 |
| Daudi 60 nM | 1.64E-03 | 7.0 |
| LCL1.11 10 nM | 1.13E-03 | 10.2 |
| LCL1.11 60 nM | 1.03E-03 | 11.2 |
| P493.6 10 nM | 1.50E-03 | 7.7 |
| P493.6 60 nM | 1.38E-03 | 8.4 |
| Ramos 10 nM | 1.11E-03 | 10.4 |
| Ramos 60 nM | 1.24E-03 | 9.3 |

| Cell line & incubation solution | $k_d$ (1/s) | half-life (min) |
|---|---|---|
| Daudi FITC-Fab | 1.66E-03 | 7.0 |
| Daudi FITC-Fab + unl Fab | 1.16E-03 | 10.0 |
| Ramos FITC-Fab | 1.23E-03 | 9.4 |
| Ramos FITC-Fab + unl Fab | 1.25E-03 | 9.2 |
| P493.6 FITC-Fab | 1.40E-03 | 8.3 |
| P493.6 FITC-Fab + unl Fab | 1.22E-03 | 9.5 |
| LCL1.11 FITC-Fab | 1.16E-03 | 10.0 |
| LCL1.11 FITC-Fab + unl Fab | 1.42E-03 | 8.1 |

**Suppl. Table 2.** Dissociation rate constants and half-lifes for RTX-Fab.
